# Ultrasound and Thermosonication as Promising Technologies for Processing Plant-Based Beverages: A Review

**DOI:** 10.17113/ftb.62.04.24.8624

**Published:** 2024-12

**Authors:** Dalia M. Sotelo-Lara, Genaro G. Amador-Espejo, Diego F. Álvarez-Araiza, Alondra K. Cordero-Rivera, Karla G. Millán-Quintero, Rocío Campos-Vega, Rita M. Velázquez-Estrada

**Affiliations:** 1 National Technological Institute of Mexico / Technological Institute of Tepic, Av. Tecnológico 2595, Col. Lagos del Country, C.P. 63175, Tepic, Nayarit, Mexico; 2 CONAHCYT-IPN Applied Biotechnology Research Center, Ex-Hacienda San Juan Molino Carretera Estatal Tecuexcomac-Tepetitla Km 1.5, C.P. 90700, Tlaxcala, Mexico; 3 Graduate Program in Food from the Center of the Republic (PROPAC), Research and Graduate Studies in Food Science, School of Chemistry, Universidad Autónoma de Querétaro, Centro Universitario, Cerro de las Campanas S/N, Santiago de Querétaro, Querétaro C.P. 76010, Mexico

**Keywords:** plant-based beverages, ultrasound technologies, physicochemical properties

## Abstract

Plant-based beverages are water-soluble extracts of cereals, pseudocereals, seeds and legumes that resemble milk in appearance. However, these products have important differences compared to normal liquid milk, such as nutritional composition, sensorial properties and shelf-life stability. Increasing number of consumers are opting for these beverages due to lactose intolerance, milk protein allergies or lifestyle. In this regard, different emerging technologies have been investigated to solve problems such as shelf life, nutritional and emulsion stability as well as sensory acceptability, without using high temperatures since heat treatments decrease the content of some bioactive compounds. Ultrasound technology alone or combined with temperature (thermosonication) could be a valuable tool to improve the properties of plant-based beverages. Therefore, this review provides a detailed analysis of the effect of ultrasound and thermosonication on the physical, bioactive, microbiological and sensory properties of almond-, soybean-, coconut-, hazelnut- and peanut-based beverages, among others.

## INTRODUCTION

The demand for new food products of natural origin has increased due to shifts in consumption patterns and growing health concerns associated with specific products (*1*). One such category that is gaining popularity is vegetable beverages, often referred to as plant-based "beverages", derived from sources like soybean, rice, almonds, peanuts or oats. These products face considerable challenges in maintaining physical stability (*2*).

They have garnered attention among consumers pursuing healthier lifestyles because of their rich content of phenolic compounds, unsaturated fatty acids and bioactive compounds such as phytosterols and isoflavones (*2*). Another notable trend in food consumption is the clean label movement, which advocates for reducing additives in products (*3*). This has prompted the food industry to explore processing techniques that minimize or eliminate the use of stabilizers, antimicrobials and similar additives.

In the case of plant-based beverages, stabilizers like carrageenan or thickener gums are commonly used to prevent phase separation and sedimentation (*4*). However, the traditional heat treatments applied during food processing can significantly degrade the bioactive compounds of beverages. Various non-thermal technologies such as high hydrostatic pressure, pulsed electric field (*5*), microwave (*6*), irradiation (*7*) and ultrasound (*8*) have been developed to address this issue. These technologies aim to preserve nutritional and bioactive molecules more effectively (*9*, *10*). Non-thermal treatments have shown promise for plant-based beverages such as almond (*11*), soybean (*12*) and maize (*13*), demonstrating minimal impact on particle size and physical stability.

Ultrasound, defined as pressure waves with frequencies above 20 kHz, induces the formation of microbubbles through cavitation within the food system (*8*, *14*, *15*). Combining ultrasound with moderate heat (50-60 °C), also known as thermosonication, has been effective in microbial inactivation and reducing enzymatic activity (*8*, *14*, *15*). Furthermore, ultrasound has shown potential to reduce particle size and enhance physical stability (*16*).

Despite significant research on ultrasound in different food processing areas, such as juices and nectars (*8*, *10*, *17*), comprehensive reviews on its impact specifically on plant-based beverages remain limited (*15*). Therefore, this review focuses on the current research into the ultrasonic and thermosonic processing of plant-based beverages. It covers consumption trends, market growth and the effects of ultrasound and thermosonication on the physical, bioactive, microbiological and sensory properties of beverages such as almond, soybean, coconut, hazelnut and peanut. Finally, it addresses future challenges and research directions in this evolving field.

## PLANT-BASED BEVERAGES AND CONSUMPTION TRENDS

Plant-based beverages are defined as aqueous plant extracts that have similar sensory properties to regular fluid milk (*15*). Beverages can be made from cereals (oats, rice and maize), legumes (soybeans and peas), pseudo-cereals (amaranth and quinoa), seeds (peanuts, sesame and sunflower), nuts (walnuts and almonds) and high-protein or fatty fruits (coconut). Plant-based beverages are obtained by the solid-liquid extraction of raw materials using mechanical force. Breaking down the plant matrix makes it easier to obtain phytochemical and hydrocolloid compounds from it. However, these extracts tend to have large, insoluble and unstable particles (*2*), which is why they are not physically stable.

According to the data collected by Grand View Research (*18*), the consumption of plant-based beverages (cow’s milk substitutes) based on almonds, soybeans, oats and nuts has increased worldwide by 33.5 % in the last five years. This market was valued at USD 26.80 billion in 2022 and is expected to reach a compound annual growth rate of more than 13.1 % from 2023 to 2030 (*18*).

Soybean-based beverages have been the most consumed plant-based beverages in key markets; however, oat as a raw material was the most sold product from 2019 to 2021 and other sources such as almonds, coconut, rice and soybeans, among others, have been used to a lesser extent. It should be noted that these market reports are mainly from the United States, United Kingdom, Argentina, Belgium, Brazil and Mexico (*19*). It is estimated that North America will have the largest revenue in the global market of vegetable beverages, although Asia is expected to be the fastest growing region, including countries such as China, India and South Korea (*20*).

Plant-based beverages, a sector of important growth in the food industry, are widely available in local supermarkets worldwide, where the consumer brands that stand out are DREAM, Danone, Daiya Foods, Archer Daniels Midland, Malk Organic, Ripple Foods, The New Barn and Califia Farms (*20*). It is important to highlight that various authors have associated the increase in the consumption of these plant-based beverages with increased environmental awareness, a preference for low-calorie products, specific diets (veganism) and medical reasons, including an intolerance to lactose or hypercholesterolaemia (*19*, *20*).

### Lactose intolerance

The National Institutes of Health Consensus Conference defined lactose intolerance as a gastrointestinal symptom that occurs in a person with lactose dyspepsia after consuming a single dose of lactose that is not seen when the person takes a placebo (*21*).

The worldwide prevalence of confirmed cases of lactose intolerance is approx. 57 %. The true prevalence, however, is estimated to be over 65 % and the distribution of cases globally is very uneven, with different incidences in different regions of the world (*22*). Lactose intolerance is a clinical condition characterised by symptoms attributable to lactose malabsorption such as pain and abdominal distention, flatulence and diarrhoea that occur after lactose consumption. The symptoms of lactose intolerance appear when lactase activity decreases by 50 % (*21*-*23*).

### Cow’s milk protein allergy

Food hypersensitivity or allergy has become a growing global health problem, causing socioeconomic concerns and affecting the quality of life of consumers. Food allergy is defined as an abnormal response of the immune system to the presence of an allergenic protein (*24*). Any protein can trigger an allergic reaction. There are more than 170 allergenic foods. However, in 2020, the Food and Agriculture Organization of the United Nations (FAO) classified cow's milk, eggs, shellfish, shrimp, peanuts, tree nuts, wheat and soybeans as the top eight allergenic foods (*24*, *25*).

Cow’s milk protein allergy (CMA) is the most common food allergy in children under 6 years of age, with a prevalence of 5-15 %. CMA can be defined as any adverse reaction caused by immunological mechanisms mediated by IgE against one or more milk proteins (*26*, *27*). Cow’s milk contains approx. 20 proteins with sensitising potential distributed in the whey and casein fractions; the most important of these are β-lactoglobulin, α-lactalbumin, casein allergens and immunoglobulins. CMA is more common in infants because their digestive system is not yet able to process these types of proteins. However, this condition is also observed in adults (*28*).

### Veganism

In recent years, there has been an increase in the consumption of vegan diets because of their associated health benefits. These effects include the reduction of the risk of cardiovascular diseases, body mass and cancer and the prevention and/or treatment of type 2 diabetes, among others (*29*-*32*).

In this sense, it is estimated that the number of people eating a plant-based diet has increased. The Plant-Based Foods Association (PBFA) reported that 70 % of people in the United States consumed plant-based foods in 2023, compared to 66 % in 2022 (*33*). Additionally, the plant-based food market is expected to be worth $22.3 billion by 2029, with an expected compound annual growth rate of 11.82 %.

Furthermore, some authors have labelled the vegan diet as the ’most ethical’ due to its benefits for animal welfare. Vegans are characterised by the fact that they do not use any product that contains any element of animal origin or in which animals have been used for manufacturing processes, be it clothing, pharmaceuticals, cosmetics or food (*32*, *34*, *35*).

Due to health concerns and increasing trends in dietary choices, there is a shift towards more dairy-free products such as probiotic fermented cereals, dairy-free milk replacers and fruit and vegetable beverages (*36*-*38*). In this regard, unconventional technologies that have a potential to provide a plant-based product as an alternative to cow's milk could be an option.

## ULTRASOUND AND THERMOSONICATION

The development of non-thermal technologies takes the relationship between food, diet and health into account and aims to use natural ingredients, improve quality and performance, provide functional stability and reduce energy consumption. These technologies include pulsed electric field, high hydrostatic pressure, irradiation, oscillating magnetic field, cold plasma and ultrasound (*8*, *39*, *40*).

**Fig. 1:**
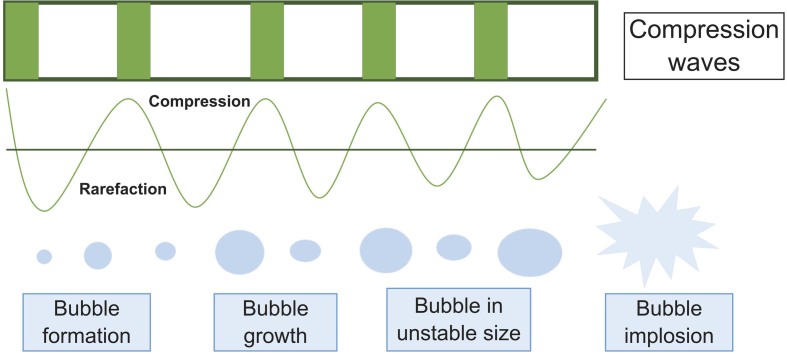
Ultrasonic cavitation phenomenon

High-intensity ultrasound is a technology that is commonly used in the food industry. Its main effect is based on the phenomenon of acoustic cavitation, which occurs when ultrasonic waves penetrate a liquid medium and change the pressure, causing the liquid to drop below vapour pressure and bubbles to form. The cavitation bubbles are formed by gas nuclei dissolved in the liquid medium, which begin to grow due to the compression and decompression of the high-intensity waves until they reach a critical size that causes their collapse (*41*). When cavitation bubbles implode, they generate localised energy accumulation, resulting in regions of very high pressure and temperature that produce waves of shear energy and turbulence (*15*, *16*, *42*) (Fig. 1).

The localised sterilisation zones created by ultrasound treatment can reach temperatures of 5000 K and pressures of 100 MPa in microseconds, which can inactivate microorganisms. Cavitation can also lead to the formation of hydroxyl radicals and hydrogen atoms, generated in response to water vapour that sonochemically disintegrates when the bubbles collapse. These substances play a major role in killing microbes because they contain free radicals and electronically excited species that cause the damage. Furthermore, oxidative compounds can cause sublethal damage to cell walls (*43*). To produce a sterilised product, a combination of sonication and heat was used in a process called thermosonication (*11*, *43*).

Thermosonication is defined as an ultrasound treatment accompanied by a moderate temperature treatment (45–60 °C). It has been shown that this method eliminates microorganisms faster than ultrasound alone, and in some cases, thermosonication has worked well enough to be used instead of thermal pasteurization for juices with inactivation around 4 log CFU/mL in most cases (*44*-*46*). Although most studies have tested thermosonication on fruit juices and nectars (*8*), it has recently shown positive results on the quality of different beverages based on cereals and seeds. In this sense, Fahmi *et al.* (*47*) showed an increase in isoflavones, aglycones and glycosides from a soybean beverage. Similarly, bioactive compounds were released in an almond beverage and thus maintained its sensory appeal (*48*).

Different reviews have described ultrasound equipment and mechanisms used in food production. In this sense, it is important to take some of the new applications of this technology into account, which will be discussed in the following sections.

## PLANT-BASED BEVERAGES TREATED BY ULTRASOUND/THERMOSONICATION

**Fig. 2:**
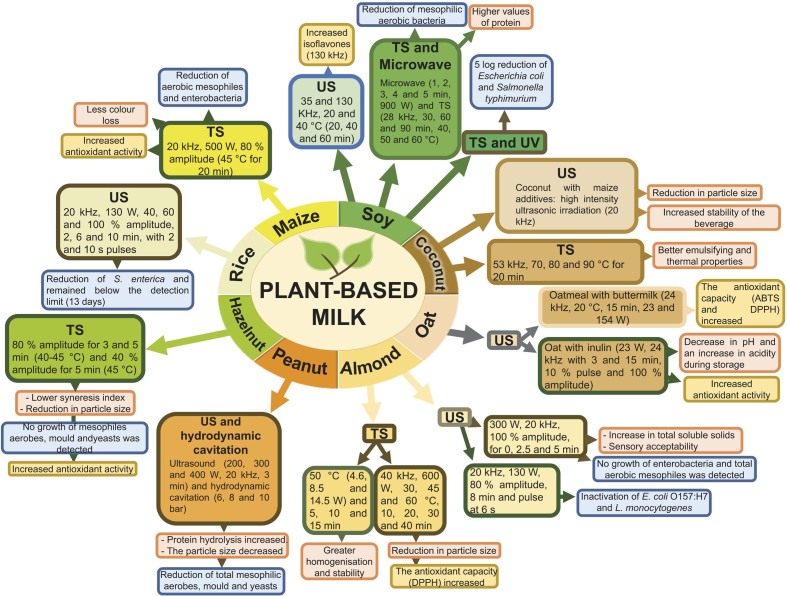
Impact of green technologies on plant-based beverages (US=ultrasound, TS=thermosonication, UV=ultraviolet light)

Plant-based beverages are derived from different plant sources and each plant raw material has unique properties in terms of taste, texture and nutritional composition. The use of ultrasound for plant-based beverages has been studied less than for juices or nectars. In this section a description of several plant-based beverages treated by ultrasound or thermosonication is included. Also, information from different studies is shown in Fig. 2 and Table 1 (*11*-*13*, *43*, *47*-*58*).

**Table 1:** Application of different treatment conditions and their effect on plant-based beverages

Beverage	Treatment condition	Treatment effect	Reference
Almond	Ultrasound (300 W, 20 kHz, 100 % amplitude for 0, 2.5 and 5 min). Sonotrode of 13 mm	Decrease in total soluble solids and colour changes (L* and b*) in some samples. No growth of microorganisms. Longer treatments result in less sedimentation of particles. Pseudoplastic behaviour. A fivefold decrease in the particle size. Adding sugar, stevia and rose water (8.05, 0.55 and 6.38 %, respectively) was suggested for the best formulation.	(*49*)
Almond	Ultrasound (20 kHz, 130 W, 2 and 8 min, 2, 6, 4 seconds pulse)	A reduction of 3.81 and 4 log CFU/mL of *E. coli* and *L. monocytogenes*, respectively (initial load of 5 log CFU/mL).	(*50*)
Almond	Thermosonication (600 W, 40 kHz, 45 and 60 °C, 10, 20, 30 and 40 min)	Increased browning index, increased turbidity and reduced particle size with no effect on pH and total soluble solids. Lipoxygenase and peroxidase residual activity of 5.12, and 6.34 %, respectively, at 60 °C for 40 min. Complete inactivation of yeast, mould and aerobic mesophilic bacteria after thermosonication (45 °C for 40 min). Higher content of bioactive compounds and antioxidant activity. Increased viscosity with higher temperatures and longer duration. Thermosonication can severely affect sensory scores at higher temperatures and longer duration.	(*11*)
Almond	Thermosonication (100, 200 and 300 W, 19 kHz at 50 °C for 5, 10 and 15 min). Sonotrode of 13 mm	Decrease in pH without formation of hydroperoxides but with degradation of fatty acids. Thermosonication at 50 °C at 14.5 W could favour non-enzymatic browning. Thermosonication treatments at high acoustic power favoured better colour results. Thermosonication causes the loss of some ethyl ester acids. Reduction of only 10 % of phenolic compound content, an increase of total flavonoid content and antioxidant activity.	(*48*)
Soybean	Thermosonication (35 and 130 kHz, 20 and 40 °C, 20, 40 and 60 min)	Higher increase of isoflavones, glycosides and aglycones at 35 than at 130 kHz. Higher content of isoflavones, glycosides and aglycones with longer sonication time and higher temperature.	(*47*)
Soybean	Ultrasound (25 kHz, 400 W, 1-16 min) and microwave (2450 MHz, 70-100 °C, 2-10 min)	Trypsin inhibitor reduction of 52 % with thermosonication (16 min) and 84 % with microwave (100 °C for 10 min), the latter having an *in vitro* protein digestibility of 84.03 % in 16-minute treatment.	(*51*)
Soybean	Thermosonication (28 kHz, 30, 60 and 90 min, 40, 50 and 60 °C) and microwave (1, 2, 3, 4 and 5 min, 900 W)	There is no pH difference. Temperature and time of thermosonication affect product colour. Microwave reduces protein removal, while adequate thermosonication can attenuate the effect of microwave. FTIR confirmed conformational deformation of proteins. Increased fat content with microwave and thermosonication treatments. Lipoxygenase is sensitive to microwave treatment and trypsin is sensitive to thermosonication. Thermosonication and microwave-assisted treatment enhanced microbial reduction. Increase in viscosity of 68 % after thermosonication (60 °C for 90 min).	(*12*)
Soybean	Ultraviolet light with thermosonication (60 and 105 °C, 10 min and flow rate of 75 mL/min)	Quality parameters (pH, protein and colour) remain unchanged. Increase of ɑ-dicarbonyl compounds (glyoxal and methylglyoxal) and advanced glycation end products (Nε-(1-carboxymethyl)-l-lysine and Nε-(1-carboxyethyl)-l-lysine) with heat treatment. Reduction of 5 log in *Escherichia coli* and *Salmonella typhimurium*.	(*52*)
Coconut	Ultrasound irradiation (20 kHz)	Colour parameters (L*, a*, b* and Δ*E*) and surface tension did not change. Pseudoplastic behaviour. Particle size reduction of fat globules with an increase in ζ-potential.	(*53*)
Coconut	Thermosonication (53 kHz, 70, 80 or 90 °C for 20 min)	Better emulsifying and thermal properties, higher solubility and surface hydrophobicity, and lower amount of free sulfhydryl groups. Change in the secondary structure of the protein, higher ζ-potential and viscosity, as well as an increase in particle size.	(*54*)
Hazelnut	Ultrasound (40 and 60 % amplitude for 5, 10, 15, 20 and 25 min. Also, 80 % amplitude for 3, 5, 10 and 15 min). Sonotrode of 13 mm	Slight decrease in pH. Decrease in the colour parameters (L* and b*), particle size and soluble protein content. Complete reduction in aerobic mesophilic bacteria, yeast and mould (80 % for 15 min). Increase in phenolic compounds and antioxidant activity in all treated samples with lower syneresis.	(*43*)
Maize	Thermosonication (20 kHz, 500 W, 80 % amplitude, 45 °C for 20 min). Sonotrode of 18 mm	There is no difference in pH or titratable acidity. There is an increase in total soluble solids in the white maize beverage and a decrease in the purple maize beverage. Reduction of bacteria (mesophilic aerobic 2.59 log CFU/mL and enterobacteria 2.39 log CFU/mL). Inactivation of yeasts, moulds and spores. Increased antioxidant activity with higher concentrations of ferulic and chlorogenic acids, but decreased phenolic compounds. Pseudoplastic behaviour in both beverages, with no differences in the consistency coefficient (*K*) or flow index (n).	(*13*)
Peanut	Ultrasound (200, 300 and 400 W, 20 kHz, 3 min) and pressure (6, 8 and 10 bar)	Higher total soluble solids content, *ζ*-potential and pH with a decrease in titratable acidity. Increased a* and h* values and decreased b* and C* values in ultrasound- and pressure-treated samples. Microbial reduction (1.53 log CFU/mL in aerobic mesophiles, about 2 log CFU/mL in yeast and moulds). Non-Newtonian behaviour by decreasing particle size with ultrasonic intensities (with a better sedimentation rate) and higher pressures.	(*55*)
Whey and oat	Ultrasound (24 kHz, 0, 3 and 10 min, 23 and 154 W)	Colour, titratable acidity, composition and pH without significant differences. Higher antioxidant activity in the ultrasound-treated samples, with higher ABTS content.	(*56*)
Whey and oat	Ultrasound (23 W, 24 kHz, 3 and 15 min, 10 % pulse and 100 % amplitude)	Decrease in pH and increase in antioxidant activity and acidity during storage. Inhibitory effect of angiotensin-converting enzyme activity higher than 50 %. Better acceptance of ultrasound-treated beverages than heat-treated ones.	(*57*)
Rice	Ultrasound (20 kHz, 40, 60 and 130 W, 100 % amplitude, 2, 6 and 10 min, with 2 and 10 s pulses)	Reduction of *Salmonella enterica* ATCC 35664 (3 and 1 log CFU/mL), remaining below the detection limit during storage (13 days at 4 °C).	(*58*)

### Almond (Prunus dulcis)

The consumption of almond beverages has increased worldwide, but it is still low compared to soybean. In this sense, only a few studies have been carried out in the last eight years. Maghsoudlou *et al.* (*49*) were the first to investigate the effect of ultrasound on almond beverages with additives like modified starch, lecithin and agar. The authors treated an almond beverage with ultrasound at 300 W, 20 kHz, 100 % amplitude and times of 0, 2.5 and 5 min. Based on the results, the authors found that there was a decrease in the total soluble solids (approx. 5.5 to 3 %) due to the partial heating of the particles or the absorption of water at high temperature, which caused the swelling of the particles. Moreover, ultrasound treatment affected the the lightness (L*) and yellowness (b*); the initial values of L* and b* were 60.4 and -1.8, respectively, while the final values were 82.6 and 1.1, respectively. The viscosity of the product was affected by temperature and showed a pseudoplastic behaviour. The flow index (n) decreased and the consistency coefficient (*K*) increased. In microbiological analysis, enterobacteria and total aerobic mesophiles were determined, and no growth of microorganisms was detected. Finally, this beverage was sweetened with sugar, stevia and rose water. The sensory analysis measured odour, colour, sweetness, bitterness and overall acceptability. The drink was well accepted by the tasters, suggesting that the use of sugar, stevia and rose water (8.05, 0.55 and 6.38 %, respectively) helped to obtain the best formulation.

In this sense, Iorio *et al.* (*50*) inactivated *Escherichia coli* O157:H7 and *Listeria monocytogenes* in almond beverage with an ultrasound treatment of 20 kHz, 130 W (80 % of amplitude, 8 min and pulse interval at 6 s for *E. coli* and 80 % amplitude, 2 min and pulse interval at 6 s for *L. monocytogenes*) and then the samples were stored at 4 °C for 2 weeks. Bacterial inactivation was close to 1 log CFU/mL in both cases. The authors argued that the improvement in shelf life could be related to sublethal injury to pathogens combined with storage under refrigeration.

Recently, Manzoor *et al.* (*11*) investigated the physicochemical and biofunctional properties of an almond beverage subjected to thermosonication treatment (40 kHz, 600 W, 30, 45 and 60 °C, 10, 20, 30 and 40 min). The application of thermosonication did not show significant effects on pH, total soluble solids or titratable acidity, but there was an important reduction in particle size measured in the particle surface area D_3,2_ and volume-weighted mean diameter D_4,3_ (from 5.22 to 3.96 µm and from 7.02 to 6.46 µm, respectively). Cloudiness was significantly reduced in the pasteurised samples, while it significantly increased in all thermosonicated samples. On the other hand, thermosonication at 40 and 50 °C improved colour parameters b* (yellowness) and L* (lightness) so that the partial precipitation of unstable particles in the suspension can result in coloured compounds, which makes the beverage more sensorially acceptable. Enzymatic analyses showed a more effective reduction of peroxidase and lipoxygenase in thermosonicated samples than in pasteurised samples. Residual lipoxygenase activity was reduced to 5.12 % when treated at 60 °C, 600 W and 40 kHz for 40 min. The peroxidase was reduced to 6.34 % with the same treatment. Total phenol content increased by 4 % at 30 °C and 6.6 % at 45 °C. On the other hand, a decrease of about 5.5 % was observed in samples treated at 60 °C. Similar results were observed for flavonols and flavonoids during thermosonication treatment at 30, 45 and 60 °C. The content of condensed tannins treated with thermosonication ranged from 205.8 to 159.2 µg/g; total antioxidant capacity (TAC), DPPH and hydroxyl radical scavenging activities also increased significantly. Moulds and yeasts as well as aerobic mesophiles were analysed, none of which were detected after treatment at 45 °C for 40 min.

With a different objective, Strieder *et al.* (*48*) performed phytochemical analysis and determined the composition of fatty acids and volatile organic compounds of thermosonicated almond-based beverages. Thermosonication was carried out at 50 °C using three acoustic powers (4.6, 8.5 and 14.5 W) and time (5, 10 and 15 min). The pH of the almond-based beverage decreased after thermosonication and the lowest values were observed in the beverage treated at 14.5 W. Similarly, a 10 % reduction in phenolic compounds was found in the treated samples compared to the control. The results of antioxidant activity were similar to the control and as for flavonoids, the samples treated at 14.5 W had lower values than the samples treated at the other acoustic powers. The content of oleic, stearic, lauric, palmitic and myristic acids in the almond beverage was determined and acoustic cavitation promoted the reduction of fat globules, resulting in better homogenisation and stability of the samples. Treatment at 50 °C with 14.5 W could promote non-enzymatic browning due to the Maillard reaction. However, the longer time and acoustic power treatments gave better colour results.

### Soybean (Glycine max)

Soybean is by far the most widely used legume for beverage production. In this regard, many studies have been carried out applying ultrasound and thermosonication. Fahmi *et al.* (*47*) applied ultrasound treatments (35 and 130 kHz, 20 and 40 °C, 20, 40 and 60 min) to soybean beverages. They used reversed-phase liquid chromatography to determine the content of isoflavones, including daidzin, genistin and their respective aglycones, daidzein and genistein. A steady increase in the content of isoflavones, glycosides and aglycones was found at both frequencies. In particular, the treatment at 35 kHz showed a more significant increase in isoflavone content than the treatment at 130 kHz. It was also observed that with the increase in sonication time (from 20 to 60 min) and temperature (from 20 to 60 °C), there was a corresponding increase in isoflavone content. This shows a positive correlation between the increase in temperature/time and a higher isoflavone content in soybean beverages; however, the frequency had an opposite effect. With all this and to confirm the behaviour of isoflavones, it is necessary to further investigate ultrasound frequencies, temperature, times and other parameters.

More recently, Vanga *et al.* (*51*) investigated the effect of ultrasound (25 kHz, 400 W, 1–16 min) and microwaves (2450 MHz, 70–100 °C, 2–10 min) on the structure of soybean beverages, *in vitro* digestibility and trypsin inhibitory activity. An incremental trend in trypsin inhibitory activity was observed with increasing duration of ultrasound treatment. Notably, the highest reduction in trypsin inhibitor content was observed at 16 min, reaching 52 %. On the other hand, the use of microwaves surpassed the effect of ultrasound, achieving a reduction of 84 %, especially at 100 °C for 10 min. Digestibility also improved, increasing to 81.38 % after just 4 min of ultrasound treatment. This trend persisted with increasing duration of treatment, eventually obtaining a digestibility percentage of 84.03 %. The effect on the protein structure was analysed. After 16 min ultrasound treatment, there was a loss of α-helix and an increase in β-sheet in the proteins, showing that ultrasound can have an effect on the molecular conformation of soybean proteins.

In this context, Kumar *et al.* (*12*) performed microwave (1, 2, 3, 4 and 5 min; 900 W) and thermosonication (28 kHz; 30, 60 and 90 min; 40, 50 and 60 °C). The authors evaluated different parameters such as the composition of proteins, fat and total soluble solids, viscosity, trypsin inhibitory activity and lipoxygenase activity in soybean beverages. The results indicated that thermosonication performed better than the ultrasound alone, but that microwave treatment gave even better results. The inactivation rate constants for the trypsin and lipoxygenase inhibitory activities were found to be 0.2034 and 0.3232 min^-1^, respectively. Lipoxygenase was more susceptible to microwave inactivation than to thermosonication, while conversely trypsin was more susceptible to thermosonication. With thermosonication, protein content, lipids, total soluble solids and viscosity increased and were affected by microwave time. The inactivation rate constants of trypsin and lipoxygenase inhibitory activities were 0.2034 and 0.3232 min^-1^, respectively. These results showed that lipoxygenase was more susceptible to inhibition by microwaves than by thermosonication. Otherwise, trypsin was more susceptible to thermosonication. Fourier transform infrared (FTIR) analysis confirmed the conformational change of the proteins and the enhancement of molecular interactions with a significant effect on viscosity (which increased up to 68 % after thermosonication treatments). Finally, the total mesophilic aerobic bacteria count showed a significant decrease.

In contrast, Park *et al.* (*52*) investigated the effect of ultraviolet light (UVC) with thermosonication on *Ecklonia cava* extract to provide an alternative process and prevent the formation of advanced glycation end products in processed soybean-based beverages. A noteworthy result was the 5-log reduction of *Escherichia coli* and *Salmonella typhimurium* in the soybean-based beverage. On the other hand, the study showed an increase in α-dicarbonyl compounds (glyoxal and methylglyoxal) and advanced glycation end products (Nε-(1-carboxymethyl)-l-lysine and Nε-(1-carboxyethyl)-l-lysine) in soybean-based beverages after pasteurisation. This finding raises questions about the possible negative effects of traditional pasteurisation methods on the nutritional quality and safety of soybean-based beverages. Furthermore, it was observed that ultraviolet light together with thermosonication, unlike pasteurisation, resulted in a decrease in α-dicarbonyl compounds and advanced glycation end products, which could be a potential advantage of the UVC with thermosonication in maintaining the nutritional quality of this type of beverages. Similarly, ultraviolet light and thermosonication of *Ecklonia cava* extract improved the antioxidant activity and preserved the quality parameters.

### Coconut (Cocos nucifera L.)

Only a few studies of the application of ultrasound and thermosonication on coconut-based products have been published. Lu *et al.* (*53*) presented the first study of a coconut-based beverage treated by ultrasound. A system consisting of a coconut emulsion (glycerol monostearate as an emulsifier) with different corn additives (corn kernels and starch with different amylose content) was tested with a high-intensity ultrasonic irradiation treatment (20 kHz). The physicochemical properties varied depending on the type of corn starch and the conditions applied. The ultrasound treatments had no effect on the colour. Emulsion stability and structural properties were analysed and compared between the emulsions. The coconut beverage with corn kernels was similar to the coconut beverage with high amylose corn starch, but the highest stability was obtained with high amylopectin content. After ultrasound treatment, it was found that the particle size was smaller than that of the untreated beverage and the particles had a monomodal size distribution. On the other hand, the electronegativity improved. However, ultrasound did not modify the rheological behaviour, which appeared as a pseudoplastic fluid.

More recently, Sun *et al.* (*54*) investigated the effect of ultrasound treatment (40 W/L, 53 kHz) combined with preheating (70, 80 or 90 °C for 20 min) on the physicochemical properties and structural characteristics of coconut globulin and coconut beverage. The ultrasound combined with preheating (90 °C) conferred improved emulsifying and thermal properties to coconut protein ande confirmed higher solubility (45.2 to 53.5 %), lower amount of free sulfhydryl groups (33.24 to 28.05 µmol/g) and higher surface hydrophobicity (7658.6 to 10 815.1). Moreover, FTIR and SEM microscopy showed changes in the secondary structure of the protein. There was also an increase in the zeta potential (-11 to -23 mV) due to the change in the physicochemical properties of the protein. On the contrary, a decrease in the thermal aggregation rate (148.5 to 13.4 %) and an increase in viscosity (126.9 to 1103.0 mPa∙s) were observed, all indicating that ultrasound combined with preheating can improve thermal stability.

### Hazelnut (Corylus avellana)

Hazelnut-based beverages are less common than other plant-based products, mainly because of the allergies associated with this nut. In this sense, the number of studies of ultrasound or thermosonication applications on hazelnut-based beverages is scarce. Atalar *et al.* (*43*) applied thermosonication treatments (80 and 40 % amplitude for 3 and 5 min, 40-45 °C) to hazelnut-based beverages. The results obtained showed a lower syneresis index (%) in thermosonicated samples than in the conventional heat treatment, suggesting an improvement of its structural properties. In terms of rheological properties, the thermosonication caused a reduction in the average particle size of the samples (about 20 % of the 80 % reported for the 5-minute treatment). The soluble protein content also decreased significantly. For colour determination, under specific treatment conditions (10 min at 40 % amplitude and 5 min at 60 and 80 % amplitude), there was a decrease in the values of L* and b*, but the value of a* did not change in the treated samples. For the microbial inactivation, total counts of aerobic mesophiles, and moulds and yeasts were 5.98 and 3.65 log CFU/mL, respectively (80 % after 15 min of treatment). An increase in phenolic compounds and antioxidant activity was also observed in all treated samples, mainly related to plant cell wall breakage, with the highest value obtained in the 60 % treatment for 25 min.

### Maize (Zea mays)

Only one study has been published on the use of thermosonication in maize-based beverages. Rodriguez-Salinas *et al.* (*13*) investigated the effect of thermosonication (20 kHz, 500 W, 80 % amplitude and 45 °C for 20 min) on maize beverages and evaluated the colour, microbiological quality and physicochemical and nutraceutical properties of white and purple corn beverages. The results showed that the lightness values (L*) between thermosonicated white and purple maize beverages showed small differences. On the other hand, a* and b* parameters increased in thermosonicated samples and tended towards the colours green and yellow. Moreover, a significant reduction in total aerobic mesophiles (from 6.96 to 4.37 log CFU/mL), enterobacteria (5.45 to 3.06 log CFU/mL) and yeast and moulds (not detected) was observed. Regarding bioactive compounds, an increase in antioxidant activity and a higher concentration of ferulic and chlorogenic acids were detected, but a decrease in phenolic compounds was observed in both beverages, without condensed tannins and a higher content of total flavonoids in the thermosonicated purple maize beverage. Finally, the viscosity of the white and purple maize beverages showed pseudoplastic behaviour according to the power law. There were no significant differences in the consistency coefficient (*K*) or the flow index (n) between the two beverages.

### Peanut (Arachis hypogaea)

As with hazelnuts and other nuts, the use of peanuts in beverages is restricted due to high number of allergies. For this reason, the number of published studies on the use of ultrasound in this type of matrix is scarce. Salve *et al.* (*55*) applied ultrasound in peanut beverages at different intensities (200, 300 and 400 W for 3 min) with increasing pressure (0.6, 0.8 and 1 MPa). The sonicated samples showed a higher total soluble solids content, higher protein hydrolysis and pH than the untreated samples. On the other hand, the treated samples showed a decrease in titratable acidity. The analysis of the ζ-potential of the ultrasonic treatments showed an increase from -27.6 to -30 mV, showing higher stability. However, an intensity of 400 W led to instability with a ζ-potential of -11 mV and resulted in the formation of aggregates. The particle size decreased with increasing ultrasonic intensity and pressure (0.29 to 0.02 µm), while the behaviour of all samples was a non-Newtonian fluid. The samples treated with powers of 200, 300 and 400 W showed the best sedimentation index since there was no phase separation, proving that the increase in ultrasonic intensity did not affect the stability of the product. The evaluation of the colour of the samples showed no significant differences in the L* value between the ultrasound-treated and untreated beverages, except for the one treated with a power intensity of 300 W, which was slightly darker than the other ultrasound-treated samples. The colour analysis showed an increase in the a* value with different treatment methods, a decrease in the b* and C* values with increasing intensity and a slight increase in the hue value of the samples treated with ultrasound and pressure. Finally, a microbiological analysis of total aerobic mesophiles, and yeast and moulds was performed, which showed a reduction of 1.53 log and about 2 log, respectively.

### Other plant-based beverages

Another interesting group that has developed in the last few years is the beverage based on the mixture of plant and whey, which is offered as a reuse of whey (reducing its polluting effect) and containing a protein with excellent nutritional quality. It is also suitable for those who do not follow a 100 % vegetarian diet. In this way, Herrera-Ponce *et al.* (*56*) used ultrasound treatments (24 kHz; 0, 3 and 10 min; 23 and 154 W) on whey and oat (*Avena sativa* L.) beverages (50:50) to verify the quality and benefits of the products. Colour, titratable acidity and pH value showed no significant differences. In this sense, no significant differences were found in the proximate analyses (water, fat, protein, ash and carbohydrates) between the two ultrasonic powers used. However, there was a difference between the sonicated and heat-treated samples. The antioxidant capacity was higher in the sonicated samples than in the pasteurised ones, with ABTS giving better results than DPPH. In another study published by the same research group, a beverage from oat and whey containing inulin (1 and 2 %) was treated with ultrasound (23 W, 24 kHz, 3 and 15 min, 10 % pulse and 100 % amplitude) and they found a decrease in pH and an increase in acidity during storage. On the other hand, an increase in antioxidant activity was observed in most treatments. When the inhibitory effect of the angiotensin-converting enzyme activity was evaluated, it was found that all treatments were above 50 % of the inhibitory effect and that the best treatment was 1 % inulin and 15-min ultrasound with 14 days of storage with 79.63 % inhibition (*57*).

A popular food matrix in Asia that is more commonly fermented is rice-based beverages. In this sense, Campaniello *et al.* (*58*) evaluated the antibacterial effect of ultrasound treatments (20 kHz; 130 W; 40, 60 and 100 % amplitude; 2, 6 and 10 min; with 2 and 10 pulses) on the inactivation of *Salmonella enterica* ATCC 35664. The microorganism was inoculated in a rice (*Oryza sativa*) beverage at two cell counts (8 and 5 log CFU/mL). After treatment, a reduction of 3 and 1 log CFU/mL was obtained, respectively. *S. enterica* remained below the detection limit for 13 days at 4 °C.

A large number of plant-based beverages have been developed using ultrasound and thermosonication treatments and they represent an opportunity for a growing market. In all cases, the products developed were determined on a laboratory scale, which opened up the opportunity for higher production volumes, while facing the associated challenges.

## ULTRASOUND AND THERMOSONICATION EFFECTS

Ultrasound treatment can increase, decrease or inactivate various processes through specific mechanisms of action such as thermal mechanisms (generation of heat or mechanical energy) and non-thermal mechanisms (cavitation, condensation and rarefaction, formation of free radicals and micromechanical shocks), as described in the chapter on ultrasound and thermosonication. Therefore, the effects of ultrasound and thermosonication on quality (physicochemical, microbial and enzymatic) and stability (particle size, sedimentation and fatty acid reactivity) are discussed in the following section.

### Physicochemical properties

Different physicochemical properties, such as pH, particle size and colour, have been studied in plant-based beverages. Maghsoudlou *et al.* (*49*) reported an increase in total soluble solids in an almond beverage due to partial cooking of the particles or the absorption of water at high temperatures, leading to swelling of the particles. The researchers attributed the decrease in modified starch in sonicated samples to cell wall breakdown and polysaccharide hydrolysis, including modified starch, caused by the shear force due to acoustic cavitation. The titratable acidity decreased because of the change in particle charge caused by cavitation. As a result, the negative γ-potential charge increased even more, leading to an increase in pH (*55*).

Colour is an important attribute that affects consumer perception and is a visual indicator of the quality of a beverage (*15*). The scattering of particles in the beverage increases with decreasing size during ultrasonic treatment, resulting in higher light scattering and lightness values. Similar results have been found in the thermosonicated almond-based beverages, where parameters such as b* (yellowness) and L* (lightness) are improved by treatment at temperatures of 40 and 50 °C, where coloured compounds can be produced by the partial precipitation of unstable particles in the suspension, making the beverage more sensorially acceptable (*11*).

### Structural modifications

Ultrasound treatment can modify protein structure and expose some of their internal hydrophilic parts (*59*). In this sense, Režek Jambrak *et al.* (*60*) applied ultrasound treatments (20 kHz, 43-48 W/cm^2^, 15-30 min) to whey protein and found that the molecular mass of proteins decreased. The authors also observed changes in the tertiary structure of proteins, which led to an increase in charged groups (NH_4_^+^ and COO^-^) and increased water-protein interactions. Therefore, ultrasound helps to improve the water retention of beverages. On the other hand, ultrasound reduces the size of fat globules due to the implosion of bubbles generated by acoustic cavitation, which produces high-intensity shock waves (*61*). This leads to homogenisation, resulting in a higher number of fat globules per unit volume but of a smaller size. It has also been reported that the decrease in fat globule size depends on two factors: time and amplitude (*61*). The ultrasound treatment can increase the solubility of organic or inorganic salts, leading to a decrease in ash content (*62*).

### Bioactive compounds

Phenolic compounds are found in the vacuole in soluble form or attached to the cell wall as pectin, cellulose, hemicellulose and lignin (*48*). The increase in phenolic content after ultrasound treatment can be attributed to the disruption of cell walls, which could facilitate the release of the bound phenolics (*63*). An increase in bioactive compounds by thermosonication could be due to the release of secondary metabolites because the mechanical rupture of cell walls is enhanced by cavitation (*11*). Ghasemzadeh *et al.* (*64*) reported that an acoustic wave disrupts the biological cell and promotes the release of cell contents. However, higher thermosonication temperatures can reduce the content of these compounds due to thermal degradation. Moreover, the acoustic energy provided by thermosonication can lead to these increases and decreases in antioxidant capacity, as well as favour the bioavailability of bioactive compounds in the sonicated medium and promote the production of hydroxyl radicals that can oxidize phenolic compounds in the same way when high temperatures and power are used, resulting in bubble collapse (*17*).

The decrease in bioactive compounds during thermosonication at high temperatures could be associated with processing parameters including treatment time, temperature and power (*65*). On the other hand, a decrease in the formation of free radicals is associated with an increase in antioxidant activities, but a high amount of free radicals for a long time can inhibit the antioxidant activity (*65*). Similarly, the free radical scavenging activity has been reported to be lower at longer ultrasonic time and temperature, while it is higher at shorter ultrasonic time and temperature (*11*).

### Enzymatic inactivation

An enzyme is a protein whose structure can be affected by chemical (acidity, organic solvents, alkalinity, *etc*.) and physical factors (irradiation, heating, microwave, *etc*.). Its activity is determined by the degree of exposure of the active site located in the centre of the enzyme. Controlled conformational changes can increase the enzyme activity, while more severe processing conditions can reduce the catalytic activity of the enzyme (*66*, *67*).

Cavitation, the formation of free radicals and localised high temperature and pressure conditions produced by ultrasound are considered the main mechanisms causing enzyme inactivation. Sonication could cause the breaking of the hydrogen bonds and the van der Waals interactions in the polypeptide chain, which favours the modification of the secondary structure of the enzyme and consequently causes the loss of biological activity of the enzyme (*68*-*70*). This technology has been shown to have positive effects on enzyme activity by causing an acceleration of enzymatic reactions due to its physical modification method in which the formation of free radicals and shear forces act on the enzyme structure and promote interactions with the substrate (*71*, *72*).

### Sedimentation index, particle size and rheological properties

Sedimentation refers to the movement of particles or macromolecules in a field of inertia. Settling mechanisms can be explained by Stokes' law, in which the settling particle velocities and densities interact with the dispersing medium (*43*). In this case, different types of behaviour can occur. In one, the sedimentation of larger particles with different densities between the particles and the continuous phase is accelerated. On the other hand, particles with a density higher than the continuous phase will sediment over time under the force of gravity. In liquid foods such as milk, sedimentation becomes a quality problem if it is excessive (*73*).

Due to the cavitation effect, thermosonication reduces the sedimentation rate by releasing intracellular compounds that increase the mean viscosity (*43*). In peanut-based beverages treated with ultrasound (20 kHz at 200, 300 and 400 W), no phase separation was observed, which shows that the increase in ultrasound intensity does not affect the stability of the particles, as evidenced by the sedimentation index values based on the reduction in the size of the particles and thus the facilitation of intermolecular reactions. In addition, it can cause the denaturation of peanut proteins, promote the unfolding of peanut protein molecules by exposing their active sites and increase the hydrophobicity of the molecular surfaces (*55*).

On the other hand, particle size is affected because ultrasound disintegrates plant and beverage particles by low-frequency acoustic cavitation (*43*). Microbubbles are generated and constantly collapse near the interface, creating localised turbulence. The acoustic waves, high shear force generated by the microflow and high-pressure shock can cause larger droplets (*53*).

Rheology is a fundamental part of the characterisation of food products, which is defined as the science that studies the parameters of deformation of matter, which vary depending on the processing of the food transformation until the final product is manufactured (*74*). Maghsoudlou *et al.* (*49*) used the ultrasound on almond-based beverages and found that the flow index (n) decreased while the consistency coefficient (*K*) increased; this could be related to the shrinkage of the swollen starch and network destruction caused by depolymerisation, carbohydrate or thermal coagulation under ultrasonic treatment (*54*). However, thermosonication does not affect viscosity, but it may improve viscosity by aiding distribution and reducing the size of hydrocolloids (*13*).

### Microbial inactivation

Several mechanisms explain the inactivation effect of thermosonication on microorganisms, including the phenomenon of cavitation, which is able to damage the cell wall and membrane of microorganisms by forming pores (sonoporation). Sonoporation is also known as cell sonication, which creates bubbles and causes acoustic cavitation, thus modifying the permeability of the cell plasma membrane (*15*). This leads to the cell disruption and consequently to a loss of intracellular content (*14*). Ultrasound perforates the cell membranes of microorganisms and the extrusion of the intracellular matrix generates free radicals that ultimately kill the microorganisms (*75*). Cavitation also helps to inhibit the growth of microorganisms by inactivating the enzymatic activity of mitochondria (*76*). The morphology, such as the type, shape or diameter of the microorganism, affects the efficiency of microbial inactivation, making the effect of thermosonication different for different microorganisms. In this sense, Gram-negative bacteria are less resistant than Gram-positive bacteria. Due to their peptidoglycan coating, bacterial spores and moulds are more resistant to ultrasound than vegetative bacteria (*76*).

### Fatty acids

Cereals such as oats, legumes such as soybeans, walnuts, hazelnuts and almonds and some seeds (such as sunflower or sesame), which are also used for plant-based beverages, are rich in saturated and polyunsaturated fatty acids, some of which consist of more than 90 % polyunsaturated fatty acids (*39*). Lipid oxidation by thermosonication may be due to thermal and sonochemical degradation. The high temperatures generated during thermosonication and the free radicals produced may favour the oxidation of lipids. However, although the microbubbles generated by cavitation can promote lipid oxidation, short retention times and reduced heat exposure can prevent the formation of oxidizing compounds during thermosonication. On the other hand, acoustic cavitation leads to better homogenisation and stability in samples containing lipids by promoting the reduction of fat globules (*48*). It should be noted that the thermosonicated hazelnut beverage did not favour the formation of hydroperoxides, although it showed degradation of fatty acids (*48*).

Based on the microbial and physicochemical results obtained, the ultrasound application has been shown to be suitable for food processing, as it offers the possibility of combining particle reduction with microbial inactivation in one piece of equipment, which can lead to important reduction in time and cost for food processors.

## CHALLENGES AND FUTURE WORK

Today's consumers are driving the food industry to develop a range of innovative products that offer not only sensory and nutritional properties, but also functionality and affordability. The future of the beverage market will therefore depend on offering healthy lifestyle choices and taking the prevalence of food allergies and intolerances into account. In this regard, the challenge with plant-based beverages is not only in the plant-derived matrix and the extraction process, but also in the technique used to preserve them while maintaining the quality and biological functionality of the compounds. In this sense, ultrasound and thermosonication have been shown to offer several advantages for plant-based beverages. Additionally, these technologies could be useful as pre-fermentation processes for these plant-based beverages, thus producing high yielding and high-quality fermented products (*57*, *76*).

In addition, the process scaling, where equipment designers make significant improvements to increase processing volume, is probably the biggest challenge for ultrasound and thermosonication applications and their successful market entry. Nevertheless, the industry continues to operate in a niche production environment. The same line of research could also include the possible combination with other technologies such as ultraviolet light, microwave and filtration, among others. Finally, the food industry is trying to adopt environmentally friendly technologies, such as thermosonication, which are sustainable in processing by reducing energy and water consumption.

## CONCLUSION

Society's growing demand for non-dairy products and increasing competition in the food industry emphasise the potential of cutting-edge technologies like ultrasound and thermosonication as suitable replacements for traditional plant-based beverage processing methods. These technologies not only improve the quality of these beverages but also enable the production of clean-label products. Although there are no ultrasound devices that can treat large volumes, the designs show that this barrier could be broken in the near future. Additionally, because they are sustainable, they comply with environmental standards and open the door to future commercial applications that promote ecological and industrial goals.

## Data Availability

The datasets used and/or analysed during the current study are available from the corresponding author on reasonable request.
